# Immunoglobulin G in Ebola Outbreak Survivors, Gabon

**DOI:** 10.3201/eid1507.090402

**Published:** 2009-07

**Authors:** Nadia Wauquier, Pierre Becquart, Clélia Gasquet, Eric M. Leroy

**Affiliations:** Centre International de Recherches Médicales de Franceville, Franceville, Gabon (N. Wauquier, P Becquart, C Gasquet); Institut de Recherche pour le Développement UMR190, Marseille, France (P. Becquart, C. Gasquet, E.M. Leroy); 1 These authors contributed equally to this article.

**Keywords:** Ebola hemorrhagic fever, immunoglobulin G, viruses, Gabon, letter

**To the Editor:** Three well-documented outbreaks of Ebola hemorrhagic fever occurred from 1996 through 2001 in Gabon in central Africa (*1*). All were caused by the highly pathogenic species *Zaire ebolavirus*, which is associated with an ≈80% case-fatality rate. The first outbreak hit Mayibout, a village in northeast Gabon in January and February 1996, causing 31 cases and 21 deaths. The first victims were children who helped carry and butcher a chimpanzee carcass found in the forest. The second outbreak lasted from October 1996 through March 1997 and occurred in the Booué region, about 150 km southwest of Mayibout, Gabon. The outbreak area was located along a trunk road and railroad track, and the infection spread to several villages around Booué, then to Libreville, the capital of Gabon, where 15 cases were recorded. The third outbreak occurred October 2001 through May 2002 in the Mekambo area, about 150 km from Mayibout in the east (*2*). This outbreak consisted of several independent chains of human transmission arising from infected animal carcasses, mainly chimpanzees and gorillas. It caused 65 cases and 53 deaths and coincided with major outbreaks in great apes that decimated wild populations (*3*,*4*). A total of 207 human cases were recorded during these 3 outbreaks; 149 persons died. Of the fatal and nonfatal cases 31 and 24, respectively, were confirmed by real-time reverse transcription–PCR, antigen detection, and immunoglobulin (Ig) G ELISA at Centre International de Recherches Médicales de Franceville (CIRMF) in Gabon.

Because of the lack of available samples from survivors, little is known about the duration of IgG antibody response. However, studies of 20 survivors convalescing after the 1995 Kikwit outbreak in the Democratic Republic of the Congo (DRC) showed that *Zaire ebolavirus* IgG appeared 5 to 18 days after symptom onset and persisted at least 21 months (*5*,*6*). With the exception of 2 survivors sampled 10 years after the 1976 Yambuku outbreak in DRC (*7*), no data are available on *Zaire ebolavirus* IgG persistence beyond 21 months. Low seroprevalence rates of Ebola virus or Marburg virus found in surveys of patients in outbreak areas have been attributed to seroreversion (*8*–*10*).

To investigate the persistence of *Zaire ebolavirus* IgG, we studied laboratory-confirmed survivors of the 3 outbreaks in Gabon. The study was approved by the Gabon Ministry of Health and by the traditional chief of each village, and written informed consent was obtained from each survivor. During 3 months of investigations in the different outbreak areas beginning in June 2007, we located 11, 3, and 6 survivors of the 2001 Mekambo, 1996 Booué, and 1996 Mayibout outbreaks, respectively. During home visits, the survivors underwent a brief medical consultation, malaria smears were taken, and basic medicines were provided to the villagers. We collected blood samples in EDTA tubes; plasma was separated by centrifugation in the field and stored in dry nitrogen until transfer to the CIRMF laboratory in Gabon, where it was stored at –80°C. ELISA was performed as previously described, using reagents provided by the Special Pathogens Branch, Centers for Disease Control and Prevention (Atlanta, GA, USA) (*7*). The optical density (OD) cut-off value (0.13) was calculated as the mean + 3 SD of adjusted OD values for 103 negative control serum samples obtained from Caucasian persons living in Europe.

All 20 survivors had positive test results for *Zaire ebolavirus* IgG (Table). The adjusted OD values at a dilution of 1:1,600 ranged from 0.3 to 3.4 in the 9 survivors of the 1996 outbreaks and from 0.7 to 3.5 in the 11 survivors of the 2001 outbreak. Adjusted OD values determined during the symptomatic period and/or a few days to 1 month after recovery were available for some survivors (Table). Specific IgG appeared by day 5 after symptom onset, increased during the symptomatic period (as shown by higher titers on day 10), peaked by day 30 (2 weeks after recovery), then declined slowly over several years. *Zaire ebolavirus* IgG remained detectable, often at high levels, >11 years after the infection.

**Table:** Adjusted OD values in patients infected with *Zaire ebolavirus* during 3 outbreaks in Gabon, determined by testing at days 5, 10, and/or 30 after symptom onset and again in 2007 (7 or 11 years after recovery)*

Patient no.	Outbreak location and year	Adjusted OD at 1:1,600 dilution			
		Day 5	Day 10	Day 30	2007
1	Mayibout 1996	0.05	1.50	2.52	1.41
2	Mayibout 1996	0.44	0.58	1.14	0.30
3	Mayibout 1996	0.22	1.90		3.17
4	Mayibout 1996				2.09
5	Mayibout 1996			2.31	0.65
6	Mayibout 1996		1.11		3.46
7	Booué 1996		2.64		0.71
8	Booué 1996		1.56		0.49
9	Booué 1996	1.26	1.55		0.31
10	Mekambo 2001				2.60
11	Mekambo 2001		1.90		1.46
12	Mekambo 2001		2.05		0.84
13	Mekambo 2001		0.80		2.13
14	Mekambo 2001				1.84
15	Mekambo 2001				0.71
16	Mekambo 2001				2.77
17	Mekambo 2001	0.10			3.50
18	Mekambo 2001	0.46			1.33
19	Mekambo 2001	0.03			2.50
20	Mekambo 2001		2.90		0.99

*Blank cells indicate data are missing or testing was not performed. OD, optical density.

These long-lasting IgG antibody responses found in 20 survivors of 3 different *Zaire ebolavirus* outbreaks rule out the hypothesis that low Ebola virus (and Marburg virus) seroprevalence rates found in epidemic regions of Africa are due to rapid loss of specific IgG. Whether this immunity is sufficient to protect from recurrent infection remains undetermined. These findings show that IgG ELISA is suitable for epidemiologic and epizootiologic investigations of Ebola and that *Zaire ebolavirus* IgG is an excellent indicator of *Zaire ebolavirus* circulation in humans.
